# Ovicidal toxicity of plant essential oils and their major constituents against two mosquito vectors and their non-target aquatic predators

**DOI:** 10.1038/s41598-023-29421-2

**Published:** 2023-02-06

**Authors:** Tanapoom Moungthipmalai, Cheepchanok Puwanard, Jirapon Aungtikun, Sirawut Sittichok, Mayura Soonwera

**Affiliations:** Department of Plant Production Technology, School of Agricultural Technology, King Mongkut’s Institute of Technology Ladkrabang, Ladkrabang, Bangkok, 10520 Thailand

**Keywords:** Entomology, Plant sciences

## Abstract

Plant essential oil (EO) is a natural alternative to synthetic chemical insecticides for mosquito control. EOs from *Citrus aurantium* L., *Cymbopogon citratus* (Stapf.), and *Cinnamomum verum* (J. Presl.) were selected for topical assay of their ovicidal activity against *Aedes aegypti* (Linnaeus) and *Aedes albopictus* (Skuse). Their efficacy was compared to that of 1% (*w/w*) temephos. In addition, their non-toxicity against aquatic mosquito predators, *Poecilia latipinna* and *Poecilia reticulata*, was tested. Found by GC–MS analysis, the major constituent of *C*. *verum* EO was *trans*-cinnamaldehyde, of *C*. *aurantium* EO was d-limonene, and of *C*. *citratus* EO was geranial. Both *C*. *verum* EO and *trans*-cinnamaldehyde at a high concentration (30,000 ppm) exhibited high ovicidal activity against *Ae*. *aegypti* and *Ae*. *albopictus* eggs after 48 h of incubation with an inhibition rate of 91.0–93.0% for *C*. *verum* EO and 96.7–95.2% for *trans*-cinnamaldehyde. The combination of *C*. *verum* EO + geranial exhibited the strongest synergistic inhibition activity (100%) against the two mosquito vectors and was five times more effective than temephos. Moreover, they were not toxic to the non-target fishes. As a safe ovicidal agent for mosquito egg control, the combination of *C*. *verum* EO + geranial has excellent potential.

## Introduction

*Aedes aegypti* L. and *Aedes albopictus* (Skuse) mosquitoes that have bitten viral-infected humans transmit the disease to other humans. These mosquitoes are major vectors of arboviruses such as Zika, yellow fever, dengue, and chikungunya^1–3^. Dengue is one of the most dangerous arboviruses, causing high morbidity and mortality rates in several countries around the world including many parts of Thailand^2,4^. According to a report by the Thai Ministry of Public Health, there were 9084 and 19,380 total dengue cases in Thailand, in 2021 and 2022, respectively, with 9 and 17 fatalities, calculated into a morbidity rate of 0.06% and 0.09%, respectively^5^. Since vaccines and other drugs have limited effectiveness in controlling dengue, controlling the population of mosquitoes with pesticides is the most effective measure to avoid this contagious disease^6^.

Controlling the mosquitoes at their embryonic and larval stages is the key strategy in controlling mosquito populations^6–8^. Generally, temephos, a common synthetic organophosphate insecticide, is used extensively around the world, especially in Thailand, for killing mosquito larvae. Unfortunately, its efficacy has been reduced drastically because populations of mosquitoes have developed resistance to it^1^. Moreover, temephos incurs serious negative side effects. It causes irreversible damage to non-target aquatic predators and humans, thus limiting its use^9,10^. In this glum context, many researchers have urgently developed alternative strategies that are safer for humans and the environment^7^.

Because plant essential oils (EOs) are natural substances, they and their phytochemical constituents are good, green alternatives to temephos. They are not harmful or only slightly harmful to mammals and non-target aquatic predators of mosquitoes at a practical pesticidal level, plus they degrade rapidly in the environment^3,11^. The EOs of *Cinnamomum verum* (J. Presl.), *Citrus aurantium* L., and *Cymbopogon citratus* (Stapf.) were investigated in this study. They have been reported safe for humans and mammals as well as having low toxicity on non-target predators because they have been long used as feed in food industry as well as antibiotic and antioxidant in folk medicine for thousands of years^12–14^.

Regarding plant EOs insecticidal efficacy, they are strongly insecticidal against many insect pests, such as *Ae*. *aegypti* (Order Diptera)^15^, *Musca domestica* (Order Diptera)^16^, *Haemaphysalis longicornis* (Order Ixodidae)^17^, *Pediculus humanus capitis* (Order Phthiraptera)^18^, *Spodoptera littoralis* (Order Lepedroptera)^19^, and *Periplaneta americana* (Order Blattodea)^20^. Specifically, EOs from *C*. *verum*, *Coccinia indica*, *C*. *citratus*, *Illicium verum* (Hook.f.), and *Moringa oleifera* (Lam.) and their major constituents (geranial and d-limonene) exhibited strong ovicidal activity against many mosquitoes species like *Anopheles indica*^7^, *Ae*. *aegypti*^21–23^, and *Culex quinquefasciatus*^24^ and housefly (*M*. *domestica*)^16^ with an LC_50_ ranging from 3.31 to 303,200 ppm. Furthermore, combined formulations of EOs and EO constituents showed even higher efficacy than their individual EOs^25,26^. For example, Soonwera et al.^27^ reported that a combined *trans*-anethol + *I*. *verum* EO formulation provided complete mortality against *Ae*. *aegypti* and *Ae*. *albopictus* larvae. Another group of researchers, Andrade-Ochoa et al.^28^, showed that a combined *trans*-cinnamaldehyde + *trans*-anethol formulation and a combined *trans*-cinnamaldehyde + (–)-limonene formulation were highly insecticidal against *Cx*. *quinquefasciatus* larvae and pupae, synergistically high. Youssefi et al.^29^ stated that a combination of thymol + carvacrol provided strong ovicidal and larvicidal activities against *Cx*. *pipiens*.

To conclude, d-limonene, geranial, and *trans*-cinnamaldehyde show several dominant activities for mosquito and other insect pest control. Single and combined formulations of d-limonene showed a strong larvicidal activity against *Ae*. aegypti^25^ and also shown a strong pupicidal activity against *Ae*. aegypti and *Ae*. *albopictus*^27^. Geranial showed a strong adulticidal activity against *Ae*. aegypti, *Ae*. *albopictus*, and *M*. *domestica*^30,31^. Single and combined formulations of *trans*-cinnamaldehyde also showed a strong adulticidal activity against *Ae*. aegypti and *Ae*. *albopictus*^4,15^.

From these pieces of studies, our group was inspired to investigate the egg mortality against *Ae*. aegypti and *Ae*. *albopictus* incurred by EOs from *C*. *aurantium*, *C*. *citratus*, and *C*. *verum*, their major constituents, and several of their combinations. In addition, the biosafety of the EO treatment was evaluated against two common, non-target predators of mosquitoes, *Poecilia latipinna* and *Poecilia reticulata* fishes.

## Results

### Essential oils and GC/MS analysis

GC–MS analysis of EOs was necessary because different parts of the three plant species*—C*. *aurantium*, *C*. *citratus*, and *C*. *verum* EOs—gave different EO chemical profiles, and hence can make an accurate efficacy comparison between studies meaningless.

All EOs were pale yellow. Table 1 is a list of the components of essential oils discovered by GC–MS. The highest percentage of extraction yield, at 1.30% *v/w*, was recovered from *C*. *aurantium* EO, followed by from *C*. *citratus* EO (1.14% *v/w*), and from *C*. *verum* EO (1.01% *v/w*). *C*. *aurantium* EO, 21 chemical constituents were found to compose 96.67% of its chemical composition. d-limonene (78.15%) was the major constituent. Some other main constituents were linalool (4.80%), δ-3-carene (2.40%), and β-myrcene (2.00%). For *C*. *citratus* EO, 9 chemical constituents were found to compose 96.54% of its chemical profile. The major constituent was geranial (45.41%). A few other main constituents were neral (24.80%), 1,8-cineole (10.59%), and geraniol (4.70%). For *C*. *verum* EO contained 14 constituents as 97.26% of its chemical profile. Trans-cinnamaldehyde (73.21%) was the major constituent. Some other main constituents were benzyl alcohol (12.87%), cinnamyl acetate (2.50%), and eugenol (2.35%).

**Table 1:** Chemical composition of the essential oils *Cinnamomum verum*, *Citrus aurantium*, *and Cymbopogon citratus*.

No	Constituent^a^	RI^b^	KI^c^	Percentage of total composition			ID^d^
				*C*. *aurantium*	*C*. *citratus*	*C*. *verum*	
1	α-Pinene	949	949	1.17 ± 0.29	3.43 ± 0.06	0.80 ± 0.04	RI,MS,Std
2	Camphene	952	952	–	–	0.61 ± 0.04	RI,MS,Std
3	Sabinene	967	969	0.20 ± 0.01	–	–	RI,MS,Std
4	β-pinene	979	979	0.91 ± 0.03	–		RI,MS,Std
5	β-Myrcene	991	991	2.00 ± 0.05	–	0.52 ± 0.11	RI,MS,Std
6	α-Phellandrene	1003	1003	0.30 ± 0.07	–	0.42 ± 0.11	RI,MS,Std
7	δ-3-Carene	1006	1006	2.40 ± 0.85	–	-	RI,MS,Std
8	Benzyl alcohol	1009	1009	–	–	12.87 ± 0.69	RI,MS,Std
9	α-Terpinene	1012	1012	–	–	0.21 ± 0.09	RI,MS,Std
10	Limonene	1032	1032	78.15 ± 5.19	–	0.64 ± 0.09	RI,MS,Std
11	1,8-Cineole	1033	1033	–	10.59 ± 0.03	0.61 ± 0.06	RI,MS,Std
12	(*E*)-β-Ocimene	1050	1050	1.13 ± 0.16	–	–	RI,MS,Std
13	γ-Terpinene	1052	1052	–	0.10 ± 0.01	–	RI,MS,Std
14	Terpinolene	1089	1088	0.60 ± 0.05	–	–	RI,MS,Std
15	Linalool	1101	1101	4.80 ± 0.80	0.81 ± 0.01	–	RI,MS,Std
16	Terpinen-4-ol	1179	1179	0.17 ± 0.02	–	–	RI,MS,Std
17	α-Terpineol	1190	1191	0.92 ± 0.04	–	–	RI,MS,Std
18	Neral	1216	1216	0.75 ± 0.03	24.80 ± 4.62	–	RI,MS
19	*trans*-Cinnamaldehyde	1221	1221	–	–	73.21 ± 2.73	RI,MS,Std
20	Nerol	1233	1232	0.10 ± 0.01	–	–	RI,MS,Std
21	Geraniol	1235	1235	0.22 ± 0.08	4.70 ± 0.00	–	RI,MS,Std
22	Geranial	1246	1246	0.43 ± 0.07	45.41 ± 2.26	–	RI,MS,Std
23	Linalyl acetate	1262	1261	1.65 ± 0.32	–	–	RI,MS,Std
24	Eugenol	1355	1355	–	–	2.35 ± 0.86	RI,MS,Std
25	Neryl acetate	1368	1368	0.05 ± 0.01	–	–	RI,MS,Std
26	α-Copaene	1378	1378	–	–	1.81 ± 0.41	RI,MS
27	Geranyl acetate	1381	1381	0.19 ± 0.02	4.30 ± 0.02	–	RI,MS,Std
28	Cinnamyl acetate	1414	1414	–	–	2.50 ± 0.53	RI,MS,Std
29	Cinnamic acid	1462	1462	–	–	0.51 ± 0.19	RI,MS,Std
30	*trans*-Nerolidol	1566	1565	0.40 ± 0.01	–	–	RI,MS,Std
31	Caryophyllene oxide	1581	1581	0.13 ± 0.02	2.40 ± 0.02	–	RI,MS,Std
32	Cadalene	1657	1658	–	–	0.20 ± 0.05	RI,MS
	Total identified (%)			96.67	96.54	97.26	
	Color			Pale yellow	Pale yellow	Pale yellow	
	Yield (% *v/w*)			1.30	1.14	1.05	

^a^Constituents listed in order of elution in the HP-5MS column. ^b^*RI* Retention index calculated through the retention time in relation to the series of C_7_–C_30_ n-alkanes. ^c^*KI* Kovats retention index is taken from https://pubchem.ncbi.nlm.nih.gov. ^d^*ID* identification method: std: substance matching was done with a readily available analytical standard (Sigma-Aldrich), *RI* RI value matching with those reported in NIST 17^54^, *MS* a mass spectrum matching with chemicals in the computer mass library of Adams^53^.

### Toxicity against target mosquito

The following ovicidal activity indexes for each formulation against the two mosquito species: inhibition rate, 50% Lethal time (LT_50_), 50% Lethal concentration (LC_50_), effective inhibition rate index (EII) versus temephos are tabulated in Tables 2 and 3. The table also includes the determined synergistic index (SI) of each formulation. For example, from Table 2, *C*. *aurantium* EO at 30,000 ppm alone provided an egg inhibition rate of 78.1%, an LT_50_ of 55.3 h, and an LC_50_ of 15,071.7 h, with EII = 2.56 against *Ae*. *aegypti*. From Table 3, *C*. *aurantium* EO at 30,000 ppm alone provided an egg inhibition rate of 76.3%, an LT_50_ of 51.7 h, and an LC_50_ of 16,592.2 h, with EII = 2.59 against *Ae*. *albopictus*. Regarding the synergistic index (SI) column, since this formulation is of single *C*. *aurantium* EO, the synergistic index of combined formulation is not applicable. Several individual EOs and EO constituents at 30,000 ppm showed a significantly higher efficacy than at a lower concentration. The highest egg inhibition rate of individual EOs was at 91.0% against *Ae*. *aegypti* and 93.0% against *Ae*. *albopictus*, achieved by *C. verum* EO. At 30,000 ppm. It provided an LT_50_ of 29.7 h against *Ae*. *aegypti* and 31.1 h against *Ae*. *albopictus*. In contrast, at 10,000 ppm, *C*. *aurantium* EO provided the lowest egg inhibition rate, at 59.7%, against *Ae*. *aegypti* and 53.2% against *Ae*. *albopictus*, with an LT_50_ of 83.6 h and 85.8 h, respectively. The highest egg inhibition rate of EO constituents was 96.7% against *Ae*. *aegypti* and 95.2% against *Ae*. *albopictus*, achieved by *trans*-cinnamaldehyde, the major constituent of *C*. *verum* EO. At 30,000 ppm, *trans*-cinnamaldehyde provided an LT_50_ of 24.3 h against *Ae*. *aegypti* and 24.7 h against *Ae*. *albopictus*. In contrast, at 10,000 ppm, d-limonene, the major constituent of *C*. *aurantium* EO, provided the lowest egg inhibition rate against *Ae*. *aegypti* at 76.4% and against *Ae*. *albopictus* at 72.1%, with an LT_50_ of 39.5 h and 36.3 h, respectively. To conclude, *C*. *verum* EO exhibited a stronger ovicidal activity (lower LC_50_) against *Ae*. *aegypti*, but *trans*-cinnamaldehyde was stronger against *Ae*. *albopictus*.

**Table 2:** Ovicidal effects of EO formulations from *Cinnamomum verum*, *Citrus aurantium*, and *Cymbopogon citratus* EOs and their major constituents and combined formulations on the hatching rate of *Aedes aegypti* eggs after 48 h of incubation.

Treatment	Inhibition rate (%) ± SD	LT_50_ (h) (LL-UL)	R^2^	LC_50_ (ppm) (LL-UL)	R^2^	EII	SI	Status
*C*. *aurantium* EO 10,000 ppm	59.7 ± 4.5f	83.6 (60.6–155.8)	0.275	15,071.7 (11,735.3–18,639.5)	0.527	1.96	–	–
*C*. *aurantium* EO 30,000 ppm	78.1 ± 3.8d	55.3 (40.2–73.6)	0.338			2.56	–	–
d-Limonene 5000 ppm	36.4 ± 5.8h	41.5 (36.9–47.6)	0.581	14,604.8 (12,221.9–17,408.3)	0.711	1.19	–	–
d-Limonene 10,000 ppm	76.4 ± 2.3d	39.5 (35.4–44.8)	0.429			2.50	–	–
d-Limonene 30,000 ppm	84.0 ± 1.9cd	33.8 (24.7–40.5)	0.431			2.75	–	–
*C*. *citratus* EO 10,000 ppm	72.9 ± 2.0e	76.2 (67.6–89.4)	0.494	11,170.2 (7659.4–14,747.1)	0.339	2.39	–	–
*C*. *citratus* EO 30,000 ppm	88.0 ± 2.5c	32.1 (25.3–42.4)	0.432			2.88	–	–
Geranial 5000 ppm	40.0 ± 5.9g	36.3 (29.9–45.9)	0.443	9907.8 (7260.1–12,843.8)	0.552	1.31	–	–
Geranial 10,000 ppm	86.4 ± 3.6c	33.4 (26.5–41.1)	0.421			2.83	–	–
Geranial 30,000 ppm	90.6 ± 1.6bc	30.2 (24.7–38.5)	0.698			2.97	–	–
*C*. *verum* EO 10,000 ppm	85.1 ± 2.0cd	34.1 (27.9–43.3)	0.412	9069.4 (5126.7–12,857.9)	0.508	2.79	–	–
*C*. *verum* EO 30,000 ppm	91.0 ± 2.5bc	29.7 (25.1–33.0)	0.719			2.98	–	–
*trans*-Cinnamaldehyde 5000 ppm	49.4 ± 1.8fg	35.8 (30.0–44.6)	0.431	7205.9 (5190.2–10,276.4)	0.148	1.62	–	–
*trans*-Cinnamaldehyde 10,000 ppm	89.2 ± 5.3c	31.4 (27.2–42.1)	0.482			2.92	–	–
*trans*-Cinnamaldehyde 30,000 ppm	96.7 ± 3.9b	24.3 (20.1–29.9)	0.726			3.17	–	–
*C*. *verum* EO + geranial (2:1) 10,000 ppm	100a	17.7 (12.4–26.6)	0.881	2308.2 (982.1–3001.3)	0.319	3.28	0.29	Synergy
*C*. *citratus* EO + d-limonene (2:1) 10,000 ppm	94.5 ± 1.6b	28.6 (22.9–36.5)	0.414	4212.3 (2256.1–6101.2)	0.422	3.10	0.43	Synergy
*C*. *aurantium* EO + geranial (2:1) 10,000 ppm	81.0 ± 5.3cd	35.8 (29.8–44.6)	0.431	4306.4 (2581.1–6233.1)	0.321	2.90	0.49	Synergy
d-Limonene + geranial (1.5:1.5) 10,000 ppm	95.5 ± 5.9c	28.6 (22.9–36.5)	0.414	4200.1 (2011.0–3972.6)	0.409	3.13	0.49	Synergy
Geranial + *trans*-cinnamaldehyde (1.5:1.5) 10,000 ppm	100a	20.4 (14.1–31.7)	0.459	2901.4 (1583.4–4030.2)	0.320	3.28	0.37	Synergy
d-Limonene + *trans*-cinnamaldehyde (1.5:1.5) 10,000 ppm	100a	22.9 (17.7–30.9)	0.408	2916.0 (1905.4–4022.4)	0.331	2.66	0.26	Synergy
Temephos (positive control) 1 ppm	30.5 ± 4.6i	60.2 (51.3–77.4)	0.351			–	–	–
Ethyl alcohol (negative control)	0h	n/a	n/a			–	–	–
Water (neutral control)	0h	n/a	n/a			–	–	–
ANOVA *Df*_total_, *P* value, *F*_0.05_	239, < 0.05, n.s							

Means percentage ovicidal activities in each column followed by same letters are not significantly different by ANOVA at *P* < 0.05. *LT*_*50*_ Lethal time that kills 50% of the exposed eggs, *LC*_*50*_ Lethal concentration that kills 50% of the exposed organisms, *LL *, 95% lower confidence limit and *UL*, 95% upper confidence limit, *R*^2^ regression coefficient, *EII* effective inhibition rate index, *n.s.* not significantly different at *P* < 0.05, *SI* Synergistic index, *n/a* not available.

**Table 3:** Ovicidal effects of EO formulations from *Cinnamomum verum*, *Citrus aurantium*, and *Cymbopogon citratus* EOs and their major constituents and combined formulations on the hatching rate of *Aedes albopictus* eggs after 48 h of incubation.

Treatment	Inhibition rate (%) ± SD	LT_50_ (h) (LL-UL)	R^2^	LC_50_ (ppm) (LL-UL)	R^2^	EII	SI	Status
*C*. *aurantium* EO 10,000 ppm	53.2 ± 1.8g	85.8 (65.4–135.6)	0.154	16,592.2 (13,357.4–20,170.4)	0.541	1.80	–	–
*C*. *aurantium* EO 30,000 ppm	76.3 ± 5.3de	51.7 (39.5–71.3)	0.429			2.59	–	–
d-Limonene 5000 ppm	62.4 ± 4.4f	80.5 (70.4–95.7)	0.453	7708.9 (5590.4–9991.2)	0.835	2.11	–	–
d-Limonene 10,000 ppm	72.1 ± 1.9e	36.3 (29.9–45.9)	0.437			2.44	–	–
d-Limonene 30,000 ppm	90.8 ± 1.4bc	32.4 (27.9–43.3)	0.592			3.08	–	–
*C*. *citratus* EO 10,000 ppm	71.4 ± 3.6e	55.3 (52.0–64.9)	0.386	12,170.5 (8466.3–15,870.6)	0.314	2.42	–	–
*C*. *citratus* EO 30,000 ppm	85.1 ± 2.0c	34.6 (29.9–45.9)	0.489			2.88	–	–
Geranial 5000 ppm	81.2 ± 2.0d	73.0 (61.8–92.3)	0.374	4979.9 (3615.9–9279.7)	0.396	2.75	–	–
Geranial 10,000 ppm	81.6 ± 2.0d	34.1 (30.1–44.7)	0.421			2.77	–	–
Geranial 30,000 ppm	92.4 ± 2.0b	29.7 (28.3–39.5)	0.701			3.13	–	–
*C*. *verum* EO 10,000 ppm	88.6 ± 1.6c	41.5 (36.0–47.6)	0.475	8196.2 (4269.1–12,020.8)	0.353	3.00	–	–
*C*. *verum* EO 30,000 ppm	93.0 ± 2.5b	31.1 (28.1–40.2)	0.709			3.15	–	–
*trans*-Cinnamaldehyde 5000 ppm	83.6 ± 1.9cd	52.7 (46.4–61.8)	0.462	4530.9 (3497.5–8731.9)	0.322	2.83	–	–
*trans*-Cinnamaldehyde 10,000 ppm	86.0 ± 2.7c	30.3 (27.1–34.0)	0.592			2.92	–	–
*trans*-Cinnamaldehyde 30,000 ppm	95.2 ± 1.6b	24.7 (20.0–31.0)	0.712			3.22	–	–
*C*. *verum* EO + geranial (2:1) 10,000 ppm	100a	16.9 (13.8–25.4)	0.879	2303.5 (982.1–3001.3)	0.318	3.39	0.27	Synergy
*C*. *citratus* EO + d-limonene (2:1) 10,000 ppm	93.8 ± 3.4b	27.3 (21.5–35.2)	0.490	4255.0 (3344.5–5901.2)	0.421	3.18	0.44	Synergy
*C*. *aurantium* EO + geranial (2:1) 10,000 ppm	80.0 ± 4.7d	34.3 (29.1–43.2)	0.433	4101.5 (3650.1–6511.2)	0.398	2.71	0.26	Synergy
d-Limonene + geranial (1.5:1.5) 10,000 ppm	91.7 ± 3.8c	29.8 (23.5–34.2)	0.425	4270.4 (3451.0–3966.1)	0.409	3.11	0.48	Synergy
Geranial + *trans*-cinnamaldehyde (1.5:1.5) 10,000 ppm	100a	19.7 (13.2–29.2)	0.419	2980.3 (1478.2.4–2042.2)	0.325	3.39	0.36	Synergy
d-Limonene + *trans*-cinnamaldehyde (1.5:1.5) 10,000 ppm	100a	21.3 (17.4–31.6)	0.458	2901.2 (1815.5–3901.2)	0.339	3.39	0.47	Synergy
Temephos (positive control) 1 ppm	29.5 ± 2.4h	61.3 (51.4–78.5)	0.351			–	–	–
Ethyl alcohol (negative control)	0i	n/a	n/a			–	–	–
Water (neutral control)	0i	n/a	n/a			–	–	–
ANOVA *Df*_total_, *P* value, *F*_0.05_	239, < 0.05, n.s							

Means percentage ovicidal activities in each column followed by same letters are not significantly different by ANOVA at *P* < 0.05. *LT*_*50*_ Lethal time that kills 50% of the exposed eggs, *LC*_*50*_ lethal concentration that kills 50% of the exposed organisms, *LL* 95% lower confidence limit and *UL*, 95% upper confidence limit, *R*^2^ regression coefficient, *EII* effective inhibition rate index, *n.s.* not significantly different at *P* < 0.05; *SI* synergistic index, *n/a* not available.

Moreover, the ovicidal efficacy of all combined formulations against the eggs of the two mosquito vectors was greater than the efficacy of individual EOs and EO constituents, with a synergistic index (SI) in the range of 0.26–0.49. The highest egg inhibition rate was at 100% against both *Ae*. *aegypti* and *Ae*. *albopictus*, achieved by the combination of *C*. *verum* EO + geranial (2:1) 10,000 ppm. It provided an LT_50_ of 17.7 h against *Ae*. *aegypti* and an LT_50_ of 16.9 h against *Ae*. *albopictus*. In contrast, the combination of *C*. *aurantium* EO + geranial (2:1) 10,000 ppm provided the lowest egg inhibition rate, at 81.0% against *Ae*. *aegypti* and 80.0% against *Ae*. *albopictus*, with an LT_50_ of 35.8 h and 34.3 h, respectively. To conclude, *C*. *verum* EO + geranial (2:1) 10,000 ppm exhibited a stronger ovicidal activity against both *Ae*. *aegypti* and *Ae*. *albopictus*, in terms of low LC_50_.

Regarding the effective inhibition rate index (EII), every combination of separate EOs and EO components as well as all combined formulations showed higher than 1.0 EII—they were more toxic to the eggs of *Ae*. *aegypti* and *Ae*. *albopictus* than 1 ppm temephos was.

### Toxicity against non-target aquatic predators

The estimated LC_50_ values against *P*. *latipinna* and *P*. *reticulata*, two fish species, of all formulations are summarized in Table 4. The two species were less susceptible to individual EOs and *trans*-cinnamaldehyde, d-limonene, and geranial EO constituents than every combined formulation, in terms of LC_50_. The range of LC_50_ values against the two fish species of those EOs and EO constituents was from 8165.5 to 57,232.5 ppm, while for the combined formulations, the range was 4091.6–5921.3 ppm. On the other hand, the range for 1 ppm temephos was high toxic to both species with LC_50_ from 298.7 to 526.7 ppm.

**Table 4:** Effect of *Cinnamomum verum*, *Citrus aurantium*, and *Cymbopogon citratus* EOs and their major constituents and combined formulations against *Poecilia latipinna* and *Poecilia reticulate* fishes sharing the same ecological niche of *Aedes aegypti* and *Aedes albopictus*.

Treatment	Non-target predators	LC_50_ (ppm) (LL-UL)	Regression equation	R^2^	X^2^ (*d*.*f*.)
*C*. *aurantium* EO 10,000 ppm	*Poecilia latipinna*	18,813.3 (–)	y = − 0.399 + 0.001x	0.948	0.002 (9) n.s
	*Poecilia reticulata*	18,246.2 (–)	y = − 0.521 + 0.001x	0.967	0.004 (9) n.s
d-Limonene 10,000 ppm	*Poecilia latipinna*	40,062.4 (–)	y = − 0.153 + 0.000x	0.785	0.001 (9) n.s
	*Poecilia reticulata*	46,772.4 (28,163.8–64,304.2)	y = − 0.491 + 0.001x	0.932	0.422 (9) n.s
*C*. *citratus* EO 10,000 ppm	*Poecilia latipinna*	57,232.5 (–)	y = − 0.153 + 0.000x	0.785	0.001 (9) n.s
	*Poecilia reticulata*	51,508.8 (–)	y = − 0.153 + 0.000x	0.785	0.001 (9) n.s
Geranial 10,000 ppm	*Poecilia latipinna*	48,631.8 (33,621.4–978,426.0)	y = − 0.031 + 0.000x	0.882	3.030 (9) n.s
	*Poecilia reticulata*	36,825.6 (28,030.1–68,274.5)	y = − 0.184 + 0.000x	0.966	1.681 (9) n.s
*C*. *verum* EO 10,000 ppm	*Poecilia latipinna*	25,798.5 (13,573.1–54,386.7)	y = − 0.767 + 0.001x	0.785	0.080 (9) n.s
	*Poecilia reticulata*	25,798.5 (13,573.1–54,386.7)	y = − 0.767 + 0.001x	0.785	0.080 (9) n.s
*trans*-Cinnamaldehyde 10,000 ppm	*Poecilia latipinna*	8165.5 (–)	y = 0.135 + 0.001x	0.800	0.011 (9) n.s
	*Poecilia reticulata*	8753.5 (7541.3–10,000.9)	y = 1.344 + 0.001x	0.627	0.056 (9) n.s
*C*. *verum* EO + geranial (2:1) 10,000 ppm	*Poecilia latipinna*	5921.3 (3850.0–6953.8)	y = 1.000 + 0.001x	0.608	0.226 (9) n.s
	*Poecilia reticulata*	4832.3 (–)	y = 1.632 + 0.001x	0.499	0.038 (9) n.s
*C*. *citratus* EO + d-limonene (2:1) 10,000 ppm	*Poecilia latipinna*	4470.7 (–)	y = − 0.153 + 0.001x	0.778	0.014 (9) n.s
	*Poecilia reticulata*	4485.3 (–)	y = − 0.153 + 0.001x	0.778	0.014 (9) n.s
*C*. *aurantium* EO + geranial (2:1) 10,000 ppm	*Poecilia latipinna*	4680.2 (3550.0–6993.4)	y = − 1.000 + 0.001x	0.608	0.226 (9) n.s
	*Poecilia reticulata*	4415.4 (3921.1–7020.1)	y = 0.479 + 0.001x	0.801	2.314 (9) n.s
d-Limonene + geranial (1.5:1.5) 10,000 ppm	*Poecilia latipinna*	4554.1 (4109.5–6314.3)	y = 0.712 + 0.001x	0.844	0.147 (9) n.s
	*Poecilia reticulata*	4525.4 (3918.5–6605.6)	y = − 0.233 + 0.001x	0.983	2.550 (9) n.s
Geranial + *trans*-cinnamaldehyde (1.5:1.5) 10,000 ppm	*Poecilia latipinna*	4343.5 (–)	y = 1.632 + 0.001x	0.499	0.038 (9) n.s
	*Poecilia reticulata*	4091.6 (–)	y = 2.663 + 0.001x	0.346	0.034 (9)
d-Limonene + *trans*-cinnamaldehyde (1.5:1.5) 10,000 ppm	*Poecilia latipinna*	4343.5 (–)	y = 1.632 + 0.001x	0.499	0.038 (9) n.s
	*Poecilia reticulata*	4343.5 (–)	y = 1.632 + 0.001x	0.499	0.038 (9) n.s
Temephos 1 ppm (positive control)	*Poecilia latipinna*	526.7 (381.3–762.3)	y = 0.104 + 0.000x	0.870	3.079 (9) n.s
	*Poecilia reticulata*	298.7 (–)	y = − 0.077 + 0.001x	0.349	1.121 (9)

No mortality was observed in the control. *LC*_*50*_ Lethal concentration that kills 50% of the exposed organisms, *LL* 95% lower confidence limit, *UL* 95% upper confidence limit, *R*^2^ regression coefficient, *d*.*f*. degrees of freedom, *n.s.* not significantly different at *P* < 0.05, *n/a* not available.

On the biosafety index (BI) are shown in Table 5. All formulations provided a high BI from 1.03 to 9.77, these BIs were higher than 1. Therefore, all formulations were not toxic to both fish species.

**Table 5:** Biosafety index (BI) against *Poecilia latipinna* and *Poecilia reticulate* sharing the same ecological niche of *Aedes aegypti* and *Aedes albopictus*, exposed to *Cinnamomum verum*, *Citrus aurantium*, and *Cymbopogon citratus* EOs and their major constituents and combined formulations.

Treatment	Non-target organism	Biosafety index (BI)	
		*Ae*. *aegypti*	*Ae*. *albopictus*
*C*. *aurantium* EO 10,000 ppm	*Poecilia latipinna*	1.25	1.13
	*Poecilia reticulata*	1.21	1.10
d-Limonene 10,000 ppm	*Poecilia latipinna*	2.74	5.20
	*Poecilia reticulata*	3.20	6.07
*C*. *citratus* EO 10,000 ppm	*Poecilia latipinna*	5.12	4.70
	*Poecilia reticulata*	4.61	4.23
Geranial 10,000 ppm	*Poecilia latipinna*	4.91	9.77
	*Poecilia reticulata*	3.72	7.39
*C*. *verum* EO 10,000 ppm	*Poecilia latipinna*	2.84	3.15
	*Poecilia reticulata*	2.84	3.15
*trans*-Cinnamaldehyde 10,000 ppm	*Poecilia latipinna*	1.13	1.80
	*Poecilia reticulata*	1.21	1.93
*C*. *verum* EO + geranial (2:1) 10,000 ppm	*Poecilia latipinna*	2.57	2.57
	*Poecilia reticulata*	2.09	2.09
*C*. *citratus* EO + d-limonene (2:1) 10,000 ppm	*Poecilia latipinna*	1.06	1.05
	*Poecilia reticulata*	1.06	1.05
*C*. *aurantium* EO + geranial (2:1) 10,000 ppm	*Poecilia latipinna*	1.09	1.14
	*Poecilia reticulata*	1.03	1.08
d-Limonene + geranial (1.5:1.5) 10,000 ppm	*Poecilia latipinna*	1.08	1.07
	*Poecilia reticulata*	1.08	1.06
Geranial + *trans*-cinnamaldehyde (1.5:1.5) 10,000 ppm	*Poecilia latipinna*	1.50	1.46
	*Poecilia reticulata*	1.41	1.37
d-Limonene + *trans*-cinnamaldehyde (1.5:1.5) 10,000 ppm	*Poecilia latipinna*	1.49	1.49
	*Poecilia reticulata*	1.49	1.49

## Discussion

Regarding extraction yield, the extraction yields of all tested plants were the same or only slightly different from the corresponding yields reported by previous studies^20,32^. The extraction yield of *C*. *verum* EO was 1.05% *v/w* compared to 1.1% *v/w* found by Aungtikun and Soonweera^4^ and Soonweera et al.^20^; the extraction yield of *C*. *aurantium* EO was 1.30% *v/w* compared to 1.4% *v/w* found by Bnina et al.^33^; and the extraction yield of *C*. *citratus* EO was 1.14% *v/w*, compared to 1.2% *v/w* found by Soonwera et al.^20^. The slight differences can be attributed to many factors, e.g., the harvesting season, the integrity of the plant species, the adequate plant management, and the degree of fertility of the soil (soil chemicals and relative humidity)^20,32^.

On GC–MS analysis results, the chemical profiles of all tested EOs agreed well with those found in previous studies^13,20^. The major chemical constituent and key component (the active component^12,20^) of *C*. *verum* EO was *trans*-cinnamaldehyde (73.21% of the profile), very close to 72.2% reported by Soonwera et al.^20^; the major chemical constituent and key component^13^ of *C*. *aurantium* EO was d-limonene (78.15%), agreeing well with 73.6% supported by Bnina et al.^33^; and the major chemical constituent and key component^14^ of *C*. *citratus* EO was geranial (45.41%), agreeing well with 49.4% found by Chanthai et al.^34^. Nevertheless, some papers report larger differences. For *C*. *verum* EO, Chansang et al.^15^ reported a higher percentage of *trans*-cinnamaldehyde (90.2% compared to 73.21%); for *C*. *aurantium* EO, Zarrad et al.^32^ reported a higher percentage of d-limonene (87.5% compared to 78.15%); and for *C*. *citratus* EO, Brügger et al.^35^ reported a lower percentage of geranial (31.5% compared to 45.41%). This variation can be attributed to seasonal fluctuations, differences in temperature at the farms where these plants were cultivated, geographic location, ontogenetic variables, the growth stage of the plant at the time of harvest (pest management), and extraction method^27,32^, and all key components were robustly identified and quantified.

Regarding ovicidal efficacy results, based on LT_50_, the ranking of inhibition against *Ae*. *aegypti* and *Ae*. *albopictus* eggs was as follows: (1) *C*. *verum* EO + geranial (2:1), (2) geranial + *trans*-cinnamaldehyde (1.5:1.5), (3) d-limonene + *trans*-cinnamaldehyde (1.5:1.5), (4) *C*. *citratus* EO + d-limonene (2:1), (5) d-limonene + geranial (1.5:1.5), and (6) *C*. *aurantium* EO + geranial (2:1). All tested formulations were more effective than temephos, and one formulation was outstanding. The outstanding combination of *C*. *verum* EO + geranial (2:1), at the final concentration of 10,000 ppm each, showed the shortest lethal time (LT_50_ ranging from 16.9 to 17.7 h) and the smallest lethal concentration (2303.5 ppm for 100% mortality). In contrast, temephos showed a lethal time in the range of 60.2–61.3 h, which is longer than that (ranging from 34.3 to 35.8 h) of the least effective EO formulation, *C*. *aurantium* EO + geranial (2:1) 10,000 ppm. Previous works^4,27^ have already established the potent toxicity of several combined EOs and EO constituents against mosquitoes at most stages in their life cycle, except at the egg stage. The effectiveness of the formulations that combined EO and EO constituents and targeted mosquitoes at the egg stage of their life cycles was first reported in this study. Regarding the most effective combined formulation, it was not surprising that it was so effective since its individual components, *C. verum* EO and geranial, have already been shown to be effective against several pest insects, as presented in the following papers. Nakasen et al.^24^ supported that *C*. *verum* EO at 12.5 ppm showed high ovicidal activity against *Cx*. *quinquefasciatus* with a 100% inhibition rate and an LC_50_ of 3.31 ppm. Soonwera et al.^20^ reported that *C*. *verum* EO had strong ovicidal activity against *Periplaneta americana*. Dias et al.^36^ indicated that *trans*-cinnamaldehyde exhibited a strong insecticidal effect against *Mahanarva spectabilis* eggs. Trans-cinnamaldehyde showed a toxic effect against the eggs of *P*. *humanus capitis*^37^, and *Spodoptera littoralis*^19^. Finally, Castillo-Morales et al.^38^ reported that geranial provided strong ovicidal activity against *Ae*. *aegypti*. Regarding the low efficacy of temephos, It can be inferred that it was low because it was not designed specifically to kill mosquitoes at the egg stage but at the larval stage. The mosquito subjects were a laboratory-selected strain, not field-collected, and hence the larvae have not developed resistance to temephos. Their morbidity was confirmed. The low egg-inhibition activity of temephos is supported by previous works of Puwanard and Soonwera^23^ and Cotchakaew and Soonwera^39^, indicating that 1% (*w/w*) temephos showed an inhibition rate ranging from 9.3 to 34.6% against the eggs of *Ae*. *aegypti* and *Ae*. *albopictus*, while the EOs showed a 47.0–100% inhibition rate.

**Figure 1:**
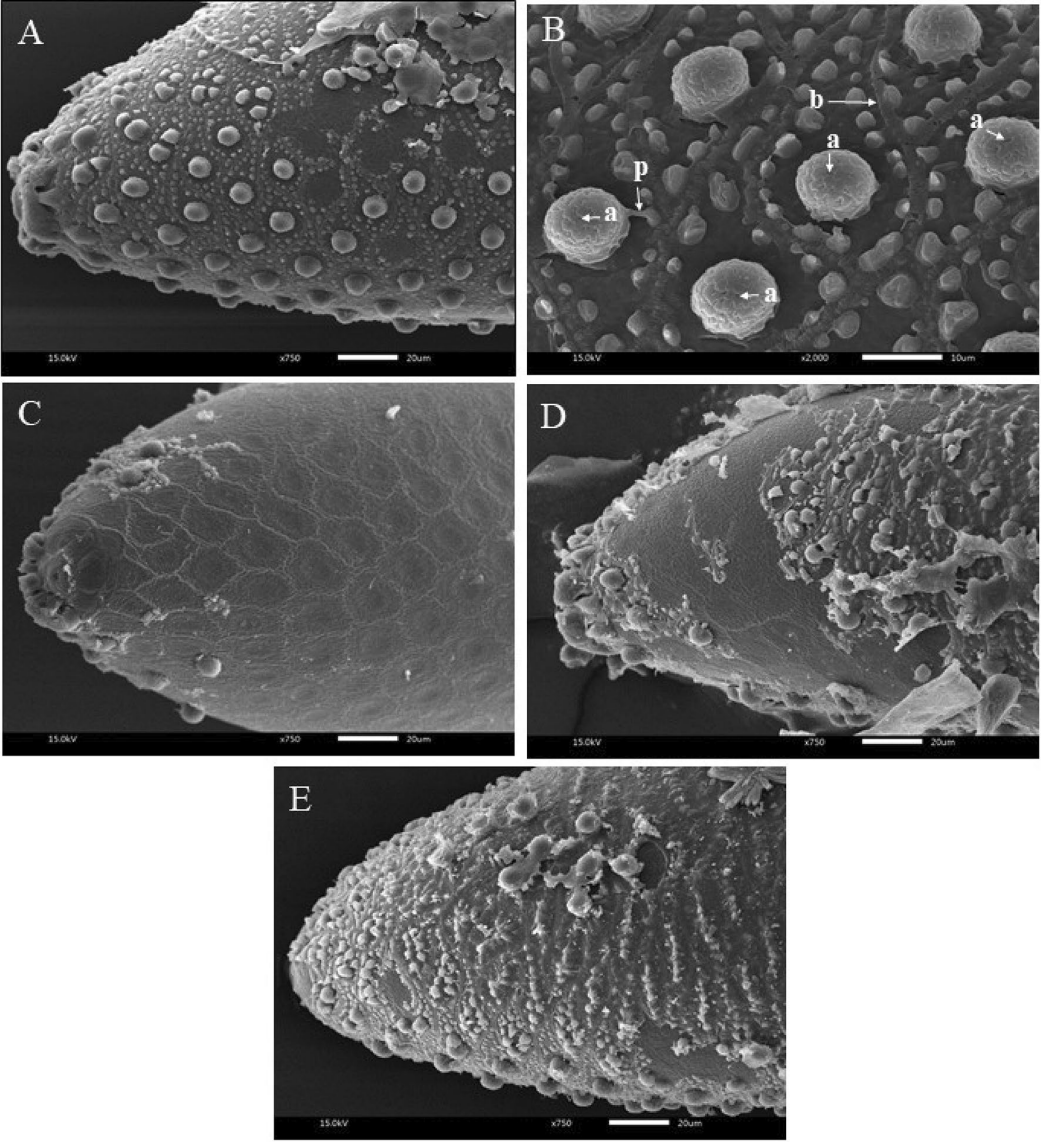
Scanning electron micrographs of *Aedes aegypti* eggs: (**A**,**B**) non-treated egg, intact exochorionic cuticle with cell borders (b), papillae (p), and aeropyles (a), morphological damage to exochorionic cuticle after treated with d-limonene (**C**), geranial (**D**), and *trans*-cinnamaldehyde (**E**).

**Figure 2:**
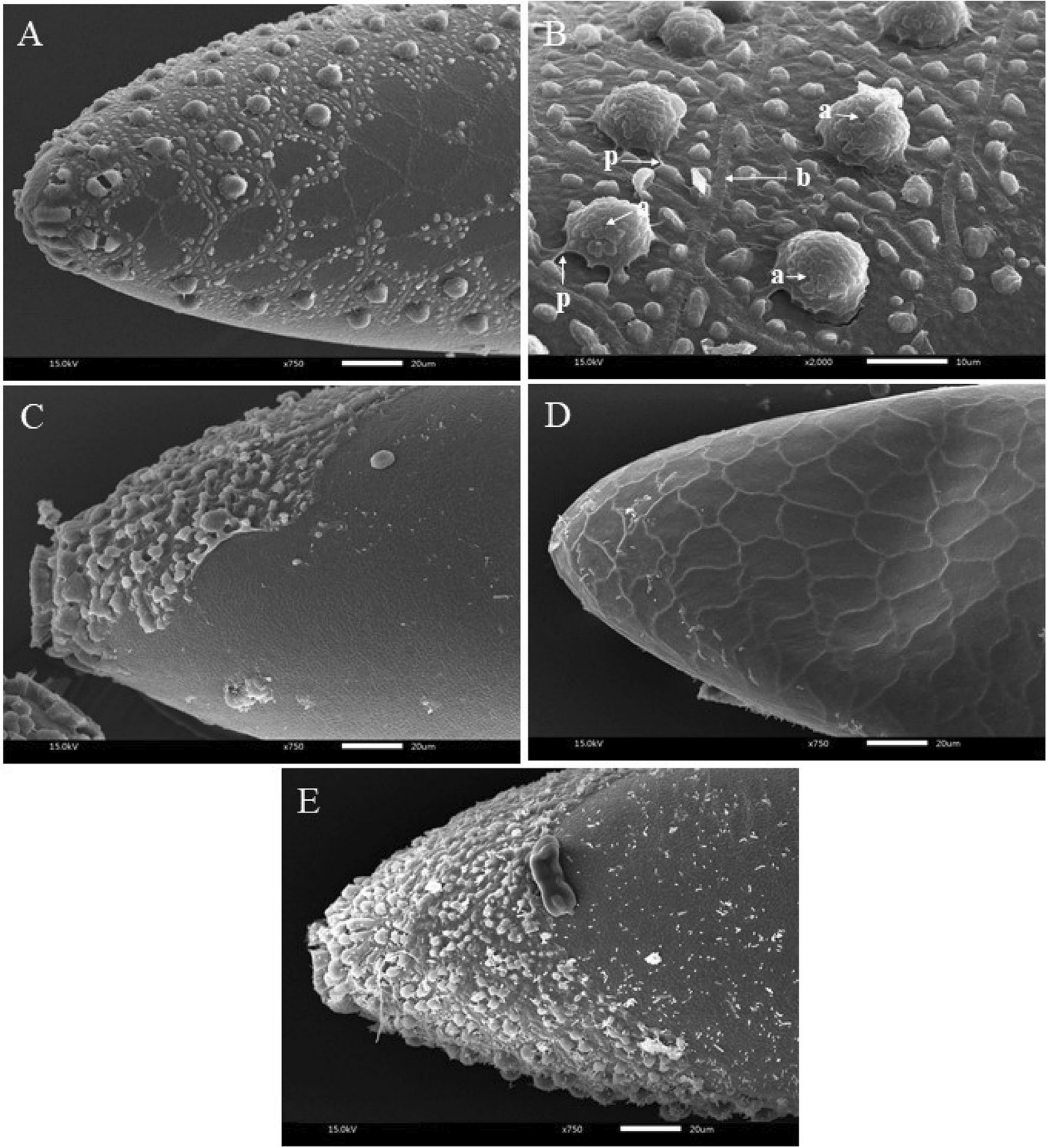
Scanning electron micrographs of *Aedes albopictus* eggs: (**A**,**B**) non-treated egg, intact exochorionic cuticle with cell borders (b), papillae (p), and aeropyles (a), morphological damage to exochorionic cuticle after treated with d-limonene (**C**), geranial (**D**), and *trans*-cinnamaldehyde (**E**).

On egg morphology, its SEM images in Figs. 1 and 2 show damages to the exochorionic meshwork and tubercles of the outer cells on exochorion cuticle (external chitin layer) with cell borders, papillae, and aeropyles^40,41^. Moreover, the cell borders and papillae as well as aeropyles were covered with a layer that was assumed to be an oil layer, which would explain the ovicidal mechanism of the EO, discussed in the paragraph below.

Regarding the mechanisms of ovicidal action, as stated in the paragraph above, the aeropyles seemed to be blocked by an oil layer, making respiration difficult or impossible. This respiration inhibition mechanism has been reported by Khedr et al.^19^ *C*. *verum* EO induced mortality of embryo and egg by forming a thin film of oil over the outer egg surface and blocking the egg respiration by sealing the aeropyles. Nakasen et al.^24^ concluded that *C*. *verum* EO destroyed chitin wall by the oil penetrating the eggshell pore leading to embryo death. Another possible mechanism of action is the mechanism that *trans*-cinnamaldehyde, the major constituent of *C. verum* EO, acts on the egg. Trans-cinnamaldehyde reduces the ATPase activity in the cell membrane of the respiratory system and inhibits the enzymes involved in cytokinesis as well as retards juvenile hormone production and cell growth in the immune system of mosquito^4,42^. In short, *C*. *verum* and *trans*-cinnamaldehyde act mainly on the respiratory system of mosquito eggs. Contrarily, geranial affects the egg's neurological system. Geranial inhibits the acetylcholinesterase (AChE) enzymes of neural cells and neuroreceptors^30^. Castillo-Morales et al.^38^ concluded that geranial penetrates through the serosal cuticle of an embryo and disturbs the embryogenesis process. Hence, the synergistic effect of the combination may stem from the fact that both substances acted along two different pathways, reinforcing one another.

**Figure 3:**
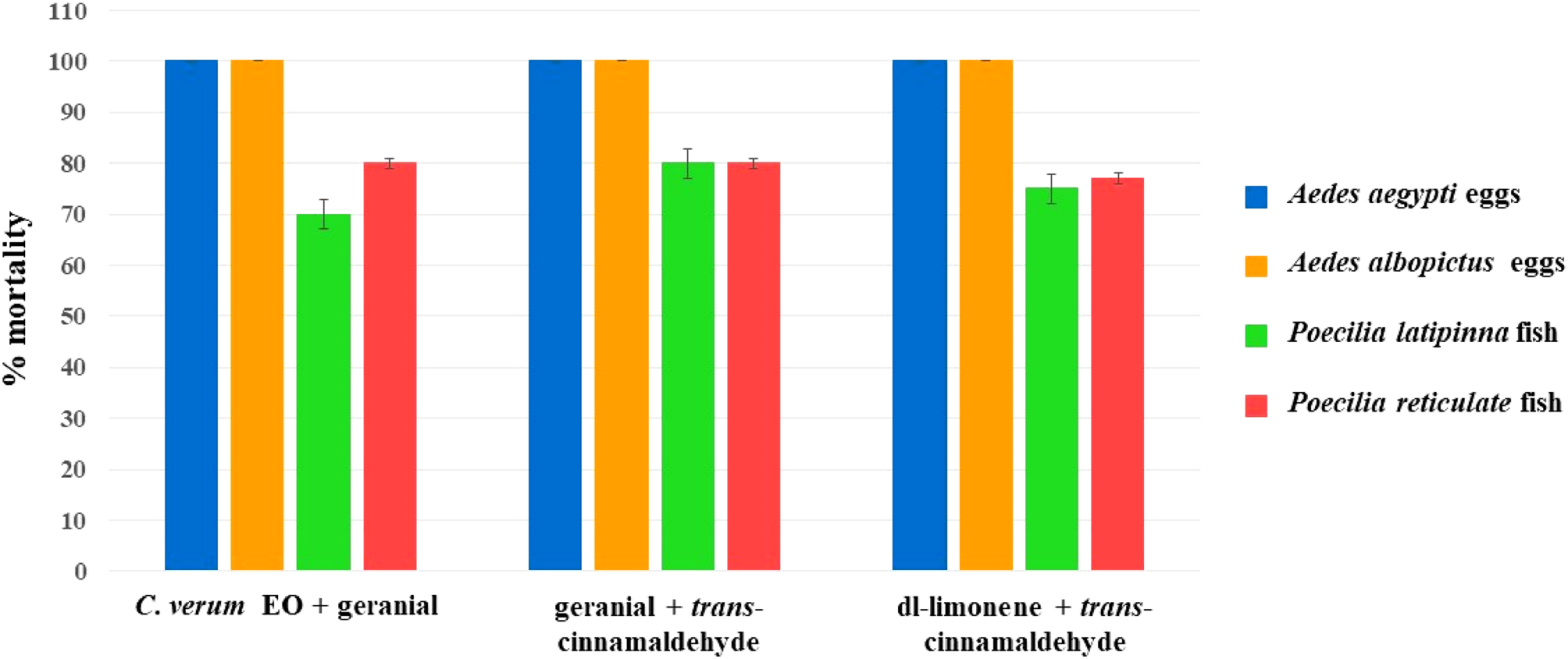
Mortality rates of the combined formulations against the eggs of *Aedes* aegypti and *Aedes albopictus*, compared to those against non-target predators of mosquitoes, *Poecilia latipinna* and *Poecilia reticulate* fishes.

On the biosafety of non-target aquatic predators of mosquito eggs, the combined EO formulation was deemed safe for *P*. *latipinna* and *P*. *reticulata*, two species of predator fish, because its BI was more than 1 and its high lethal concentration (LC_50_). EOs are also generally considered safe for other arthropods and fishes^43^. Other authors have supported the conclusion that EOs are safe for non-target organisms. Alsalhi et al.^43^ supported that *trans*-cinnamaldehyde showed very low toxicity on *Gambusia affinis* (LC_50_ = 3960.6 ppm). Nwanade et al.^17^ reported that *trans*-cinnamaldehyde provided a less toxic effect on *Tenebrio molitor* (LC_50_ = 28.4 μL/mL). In addition, Hýbl et al.^44^ indicated that *C*. *zeylanicum* EO did not show toxicity against honey bee, *Apis mellifera* (LC_50_ = 4.542 μL). Sabahi et al.^45^ reported that *C*. *citratus* EO was not toxic to *A*. *mellifera* (LD_50_ = 53,304.0 μg/mL). It has also been shown that geranial, the major constituent of *C*. *citratus* EO, had a low negative effect on a predatory bug, *Podisus nigrispinus* (LD_50_ = 25.56 μg/insect^−1^)^35^. In contrast, temephos is highly toxic to several non-target organisms e.g., *Acilius sulcatus*, *Anisops bouvieri*, and *G*. *affinis* with LC_50_ ranging from 0.957 to 4.817 ppm^43^. Chellappandian et al.^11^ reported that temephos showed a highly toxic effect against aquatic mosquito larvae predator, *Toxorhynchites splendens*. Along the same line, USA EPA^46^ concluded that temephos showed highly acute toxicity to risk quotients for freshwater fish: the LC_50_ against rainbow trout was 3490 ppb. Similarly, in this study, 1% (*w/w*) temephos showed a high level of toxicity to two fish species, *P*. *latipinna* and *P*. *reticulata* with LC_50_ ranging from 298.7 to 526.7 ppm. Furthermore, temephos resists degradation and accumulates in the environment at a high level, thus harming non-target organisms^46^. On the contrary, EOs and their constituents are natural substances that degrade quickly in the environment and do not accumulate in the environment, hence much safer for the environment. More than one BI for all formulations verified that those formulations were absolutely safe for these non-target aquatic predators. The mortality rate after the treatment of the eggs was much higher than the mortality rate against the fishes (Fig. 3). Most importantly, both EOs from *C*. *verum* and *C*. *citratus* as well as their major constituents do not exhibit cytotoxicity activity on human fibroblast cells^47,48^ and show high LD_50_ value on mammals^49,50^. They have long been used as a food ingredient, cosmetics, and folk medicine^12,14,51^. They quickly degraded in the environment^50,51^. On the other hand, temephos is toxic to the nervous systems of humans. It can cause Alzheimer’s disease as reported by Martins Laurentino et al.^52^. Because of its efficacy and safety, the combined formulation of *C*. *verum* EO + geranial should be developed as a natural insecticide for controlling the eggs of *Ae*. *aegypti* and *Ae*. *albopictus* to replace commercial synthetic insecticides. However, to develop the combined formulation into a commercial product (a spray or drops of solution into the water), it is still necessary to investigate other factors that affect to mortality of mosquito vectors and their eggs, e.g., a field study and a study of the post-application temperature effect.

## Materials and methods

### Plant collection

All plants were obtained under national and international guidelines. The plants were collected under the supervision and permission of the School of Agricultural Technology, KMITL. All of the authors complied with all local and national guidelines.

Dried barks of *C*. *verum* were purchased from a local Chinese pharmacy in Thailand (Chao Krom Poe Dispensary, Bangkok, Thailand). Peels of *C*. *aurantium* fruit were obtained from a farm in Nakhon Ratchasima province, Thailand (14° 58′ 47.6400″ N/102° 5′ 51.9756″ E). Fresh stems of *C*. *citratus* were obtained from a farm in Chanthaburi province, Thailand (12° 36′ 34″ N/102° 06′ 16″ E) in July–October 2021. All plant species were identified by Mr. Tanapoom Moungthipmalai, a herbal specialist at the KMITL herbal museum, and some of the specimens were kept at the KMITL herbal museum, School of Agricultural Technology, KMITL.

### Essential oil extraction

Plant part (1000 g) was washed and extracted by hydro-distillation in 2000 mL of distilled water at 100 °C for 5 h. The rate of distillation was two drops of EO per second. The EO was then filtered and stored in a tea color bottle at 4 °C.

### Identification of essential oil constituents through GC/MS

Samples of *C*. *aurantium*, *C*. *citratus*, and *C*. *verum* EOs were analyzed by an Agilent 6890 N gas chromatograph GC–MS at the central Laboratory, KMITL, following our previous protocol^27^. Serving as the mobile phase is 1 mL per min flow of helium (99.99%). To start, 0.2 μL of extract in ethyl alcohol solution (split ratio = 1:100) of each EO was injected into the column. A 5973-N mass spectrometer (using an HP-5 MS fused silica capillary column (30 m × 0.25 mm ID with 0.25 m film thickness of 5% phenyl-methylpolysiloxane coating), an electron ionization system with 70 eV electron energy (30–500 m z^−1^), and an Agilent 6890-N gas chromatograph (USA) made up the GC–MS system. The column temperature was programmed to increase gradually from room temperature to 50 °C and stay there for 2 min. The column temperature was then increased to 200 °C and maintained there for 3 min at a rate of 10 °C min^−1^. In the final stage, the column temperature was raised to 260 °C at a rate of 15 °C min^−1^ and held there for 20 min. The injector and detector temperature were held at 270 °C. The total running time was 40 min. A mass spectra search program with Wiley 7 N library was used for identifying all components of EOs. The mass spectra of peaks were compared with those stored in Adams^53^ and NIST 17^54^ libraries. Temperature-programmed retention indices (RI) were determined using *n*-alkanes (C_7_–C_30_). The experiment was performed in three replicates.

### Source and purity of reagents

Cinnamaldehyde (98% pure), a major constituent of *C*. *verum* EO, d-limonene (96% pure), a major constituent of *C*. *aurantium* EO, and geranial (96% pure), a major constituent of *C*. *citratus* EO together with standard *n*-alkanes (C_7_–C_30_) were supplied by Sigma-Aldrich company (USA). Temephos (1 ppm), the positive control, was obtained from Thailand’s Government Pharmaceutical Organization (GPO) (Pathum Thani, Thailand). Ethyl alcohol (95% *v/v*) was supplied by Thailand’s Liquor Distillery Organization (Chachoengsao, Thailand). All chemicals used in this study were reagent-grade.

### Insect maintenance

The mosquito eggs used in this experiment were freshly laid eggs of mosquitoes of two species, *Ae*. *aegypti* and *Ae*. *albopictus*, reared in the Entomology laboratory at the School of Agricultural Technology, KMITL. They were reared under the conditions of 26.5 ± 2 °C temperature, 75.0 ± 2% RH, and an 11 ± 13 h photoperiod. Female adult mosquitoes were fed with 2.5% glucose solution + 2.5% multivitamin syrup solution and periodically blood-fed via membrane by an artificial membrane method^1^. The first generation of eggs was used in various experiments.

### Toxicity against target mosquito

Ovicidal activity bioassay was performed on the eggs following the method of Puwanard and Soonwera^23^. The eggs used for this bioassay were stored at 26.5 ± 2 °C for 7 days after female mosquitoes had laid their eggs on a Whatman No.1^®^ filter paper. Eggs were selected under a stereomicroscope (Nikon^®^ Type 102): abnormal eggs were discarded, and normal eggs were collected for the bioassay. For each mosquito species, 25 eggs were suspended in 99 mL of distilled water in a 150 mL plastic cup. A treatment was added to the cup: 1 mL of each EO formulation. This assay was done in ten replicates for each treatment, with positive, negative, and neutral controls: 1 ppm temephos (based on the recommendation of Thailand’s Government Pharmaceutical Organization (GPO) for destroying mosquito larvae), 70% (*v*/*v*) ethyl alcohol, and pure water, respectively. The numbers of hatched larvae at 30 min, 1, 6, 24, and 48 h post-treatment were observed and recorded because it was easier and more practical to count live larvae than to count dead eggs under a stereomicroscope. The percentage inhibition rate after 48 h was determined by the formulas^23^ below,

1 $$\mathrm{Hatching\, rate }\left(\mathrm{\%}\right)= \left[\left(\frac{\mathrm{NE}}{\mathrm{NT}}\right)\times 100\right],$$

2 $$\mathrm{Inhibition\, rate }(\mathrm{\%}) = 100 -\mathrm{ hatching\, rate }(\mathrm{\%}),$$

where NE is the total number of hatched eggs and NT is the total number of eggs.

The effective inhibition rate index (EII) as a comparative efficacy index between an EO and temephos, was determined by the formula^4^ below,

3 $$\mathrm{EII }= [\mathrm{\% \,inhibition\, rate\, of\, each\, EO\, formulation}/\mathrm{\% \,inhibition\, rate\, of\, temephos}].$$

EII < 1 indicates that the EO formulation was not as effective as temephos; EII = 1 indicates that the EO formulation was as effective as temephos; and IRI > 1 indicates that the EO formulation was more effective than temephos.

Synergistic index (SI) is an efficacy comparison index between a combined formulation and the corresponding individual EO or individual EO constituent. It was calculated by the following formula^31^,

4 $${\text{SI}} = [{\text{LT}}_{{{5}0}} {\text{\,of\, combined\, formulation}}/\left( {{\text{LT}}_{{{5}0}} {\text{\,of\, individual\, EO}} + {\text{LT}}_{{{5}0}} {\text{of\, individual\, EO\, constituent}}} \right)].$$

SI < 1 indicates synergistic; SI > 1 indicates antagonistic; and SI = 0 indicates not either one.

### Toxicity against non-target aquatic predators

The experimental methods and procedures were performed in accordance with the guidelines and regulations of the National Research Council of Thailand guide for the care and use of laboratory animals and approved by the King Mongkut’s Institute of Technology Ladkrabang of animal care and use committee. This study was carried out in compliance with the Animal Research: Reporting of In Vivo Experiments (ARRIVE) guidelines.

The effect of individual EOs, EO constituents, and combined formulations against non-target aquatic predators, *P*. *latipinna* and *P*. *reticulata*, was analyzed with a modified technique reported by Rajeswary et al.^55^. The test used four concentrations (i.e., 500, 1000, 2500, and 5000 ppm) of treatment that corresponded to the estimated LC_50_ against the two mosquito species. Both fish species were purchased from a farm in Nakhon Pathom province, Thailand. They were separately kept in a glass container containing 10.5 L of water at 35 ± 2 °C and 77 ± 5% RH. With the registration number KDS2021/002 (August 2nd, 2021), the King Mongkut's Institute of Technology Ladkrabang's Ethics committee had approved each bioassay used in this study. One adult *P*. *latipinna* or *P*. *reticulata* was put in a glass jar containing 99 mL of water and contaminated with a treatment at a specified concentration. Four replicates were done for each treatment with positive control. Data on mortality and swimming sluggishness were recorded for 5 days post-treatment.

The biosafety index (BI) was determined by the formula^55^ below,

5 $${\text{BI}} = \left[ {{\text{LC}}_{{{5}0}} {\text{\,of\, non-target\, aquatic\, predators}}/{\text{LC}}_{{{5}0}} {\text{\,of\, target\, vector\, species}}} \right].$$

BI > 1 indicates that the EO formulation was safe for the non-target organisms, and BI < 1 indicates that the EO formulation was not safe for non-target organisms.

### Egg morphology and observation

After 48 h of treatment, the morphology of the external surface of the eggs of each mosquito species that underwent a treatment or control was observed under scanning electron microscopy (SEM) at the Scientific and Technological Research Equipment Centre, Chulalongkorn University, Thailand. Samples were placed in a fixative, 2.5% glutaraldehyde for 30 min in 0.1 M phosphate buffer. Thoroughly washed with the same buffer, the eggs were dehydrated by soaking in a series of alcohol solutions in water (30, 50, 70, and 95%). Each 1-h soaking process with an alcohol solution was replicated three times with an automatic tissue processor. Then, the eggs were dried with a CO_2_ critical point drier. Each dehydrated sample was mounted on a stub coated with gold–palladium and examined with a JSM-5800 LV (Tokyo, Japan) SEM. Photographs of the egg surface morphology were taken.

### Statistical analysis

The design of the experiments was completely randomized. Analysis of variance (ANOVA) and Duncan’s multiple range test at *P* < 0.05 were applied to the mortality data of mosquito eggs. The treatment time to produce 50% egg mortality (LT_50_) was determined by probit analysis. The eggs were observed at 30 min, 1, 6, 24, and 48 h after the treatment. The concentration of a treatment that provided 50% mortality (LC_50_) against mosquito eggs was determined. The tested concentrations were 10,000 and 30,000 ppm for individual EOs and 5000, 10,000, and 30,000 ppm for individual EO constituents. The LC_50_ values against the two species of fish were determined at 500, 1000, 2500, and 5000 ppm. SPSS Statistical Software Package version 22 was the statistical software package used.

## Supplementary Information

Supplementary Information.

## Data Availability

All data generated or analyzed during this study are included in this published article.

## Abbreviations

EO: Essential oil
EOs: Essential oils
BI: Biosafety index
EII: Effective inhibition rate index
SI: Synergistic index
