# Supplementary material for: Ovicidal toxicity of plant essential oils and their major constituents against two mosquito vectors and their non-target aquatic predators
Source: Sci Rep. 2023 Feb 6;13:2119. doi: 10.1038/s41598-023-29421-2 (PMC9902397; doi:10.1038/s41598-023-29421-2)
Supplement: Supplementary file 1 — Supplementary Information. [file 41598_2023_29421_MOESM1_ESM.pdf]

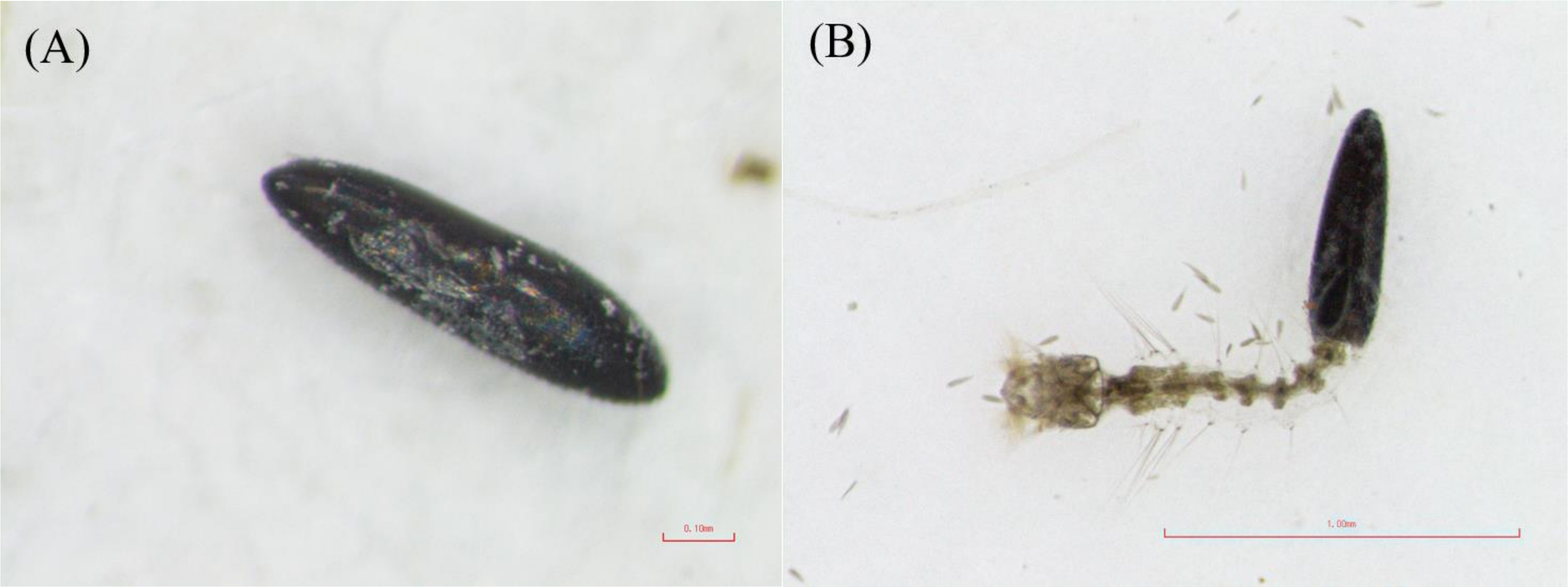

(A) unhatched egg in treatment groups, egg undeveloped and dead embryo inside the egg, and (B) development of hatched egg in control groups, showing open operculum and embryo developed into larval stage.
